# Human Split-Thickness Skin Allograft from Brain-Dead Donors

**Published:** 2016-08-01

**Authors:** A. Khodadadi, O Olang, A Makhllough, B. Nozary Heshmati, F. Azmoudeh Ardalan, S. A. Tavakoli

**Affiliations:** 1 Iranian Tissue Bank Research Center, Tehran University of Medical Sciences, Tehran, Iran; 2 Faculty of Medicine, Shahid Beheshti University of Medical Sciences, Tehran, Iran; 3 *Molecular and Biology Research Center, Faculty of Medicine, Mazandaran University of Medical Sciences, Sari, Iran*

**Keywords:** Inflammation, Symbiosis, Survival, Graft, Graft rejection, Biological dressings

## Abstract

**Background:**

Looking for an appropriate skin substitute for temporary and permanent coverage of wounds remains one of the main obstacles of medical researchers.

**Objective:**

To investigate the rate of inflammation, symbiosis, and survival of grafted allograft skin from brain-dead donors (BDDs) in rabbits.

**Methods:**

After receiving negative serologic tests of BDDs, we prepared partial thickness skin grafts. They were then used in treating wounds of 5 rabbits in comparison with split-thickness skins taken from cardiac dead donors.

**Results:**

On histopathological examinations, we found no difference between the skins. All samples were separated from the baseline in 15–20 days.

**Conclusion:**

Gamma-irradiated freeze-dried human split-thickness skin taken from BDDs is safe and can be used for the treatment of deep skin burns.

## INTRODUCTION

As long as skin loss due to different causes like major burns exists, demand for a suitable biologic dressing is outstripped the supply. Looking for various skin substitutes appropriate for temporary and permanent coverage of partial and full-thickness wounds with the lowest level of immunogenicity and rate of disease transmission remains a main concern for clinicians and tissue banks [1, 2].

The first pinch skin allograft transplantation with the use of epidermal (split thickness) skin was performed by Jacques-Louis Reverdin in 1869 [3]. Soon after, epidermal grafts usage for burn patients was introduced. In mid-forties, Medawar showed the role of immune system in graft rejection. He observed when the same transplantation was carried out a second time, the graft was rejected in half that period [4].

After invention of the first dermatome in 1939 by Padgett, removal of large flaps of skin with optimal depth became possible and a little later refrigerated skin became an optimal temporary dressing for burn wounds. Different preservation techniques of cryopreservation or preservation in glycerol were developed. In seventies cryopreserved skin allograft got its place in the field of burn and other wound care. The comparison between different methods of skin processing and its impact on efficacy has continued so far. Gaucher, *et al*’s study reconfirmed that viability of cryopreserved human skin allografts does not have negative effect on the wound healing process [5, 6].

Skin allograft, commonly used as coverage in mid or deep partial thickness burns, may be viable or nonviable depending on the way it is obtained and processed [7]. As the body cools slower than the environment, there would be some warm ischemia time, even if the body is held in a fridge. Accordingly, viable skin cannot be retrieved after 24 hours of death. It should be cryopreserved by a rate-controlled cooling apparatus to at least 80 °C after processing to be stored in very low temperature (130 °C). On the other hand, as in nonviable skin allografts cell viability is not an issue, the developed options for preservation and sterilization methods have been so wide [8, 9]. Tissue banks are looking for any feasible and practical routes to provide patients the necessary allografts for better and faster treatment.

Based on our previous experience of successful processing of skin allograft from cardiac-dead donors, processing of allografts from brain-dead donors (BDDs) has been started [1]. The current study aimed to investigate the rate of inflammation, symbiosis and survival time of grafted allograft skin as wound dressing from BDDs in rabbits to evaluate its efficacy and biocompatibility in animal models before using in burn patients.

## MATERIALS AND METHODS

To prepare a skin allograft suitable for wound dressing, it is necessary to take different steps of graft retrieval, preparation, and processing, similar to those from cardiac dead source.

Donor Screening, Skin Retrieval and Processing 

The first step in skin processing is to check medical details of donors: Five consecutive BDDs who were admitted in Organ Procurement Unit (OPU) of Iranian Tissue Bank (ITB) in the study period had blood samples for serological tests for antibodies against HIV type 1 and 2, hepatitis C virus, hepatitis B virus, and syphilis, based on national guidelines for BDDs. 

The consent for skin donation was separate from organ donation. In the case of approval of BDDs’ families for skin donation in addition to their organs, skin was retrieved after organs were removed in the operating room using an electrical dermatome with a cutting depth of 0.015 inches. The harvest was mainly from the lower limbs and buttocks not only to have a larger size grafts but also to maintain the normal body appearance for the funeral and emotional satisfaction of families. Based on ITB and the Research Center protocols, samples of 1×1 cm^2^ skin for microbial cultures were achieved at the harvesting time. The skin strips were then transported to tissue processing unit in cold conditions (4 °C) where they were placed in a two-layer sterile tank containing ringer solution mixed with antibiotics, based on ITB protocols to prevent bacterial transmission [10]. The skin was then held in antibiotic and antifungal cocktail till the reports of cultures be announced. After receiving the results of cultures, the processing phase started for tissues with negative results. Hair follicles and hypodermal fat were removed; the skin stripes were then rinsed with phosphate buffer saline (PBS) for several times. The partial-thickness skin allograft was drained and then freeze-dried with a freeze dryer (Christ Alpha 2-4, Germany) until its water content became <5%, according to the standard protocol. The final step of sterilization was gamma irradiation with 25 kGy of packaged and labeled samples. All procedures were performed in clean room conditions. 

Animals 

A total of five New Zealand white rabbits (male or female) were used for the study. They were obtained from Farabi Research Center, aged between 6 and 8 months, and weighed between 2.5 and 3 kg. All procedures were performed under the animal care guidelines. The study was approved by the Ethics Committee of Tehran University of Medical Sciences, Tehran, Iran. 

Prior to skin allograft surgery, the hair of rabbits on the dorsum was shaved. The rabbits were anesthetized with intravenous injection of xylazine (50 mg) and ketamine (250 mg in three divided doses). A deep strip of skin was taken on each side of the midline and a medium-thickness allograft skin (0.5×1.0 cm) was retrieved from BDD and cardiac-dead donors were transplanted onto the wounded areas. The four corners of each one were coaptated. All rabbits were examined on a daily basis to evaluate allograft survival and rejection time. The indices observed were color of the skin graft, marked inflammation or hyperemia, remaining in proximity to the wound, and exhibition of secretions, which indicated there was no rejection phenomenon. Darkening or swelling of the grafted skin indicated rejection of the graft; if 80% of the area of the grafted skin appeared dark or swollen, the graft was considered “necrotic,” according to previous experience. In this way, the allograft survival time could be determined. 

Histological Studies

Serial specimens after implantation of grafts were prepared for sectioning and staining by hematoxyline-eosin, which was evaluated with optic microscope.

## RESULTS

Processed Skin Allografts

**Figure 1:**
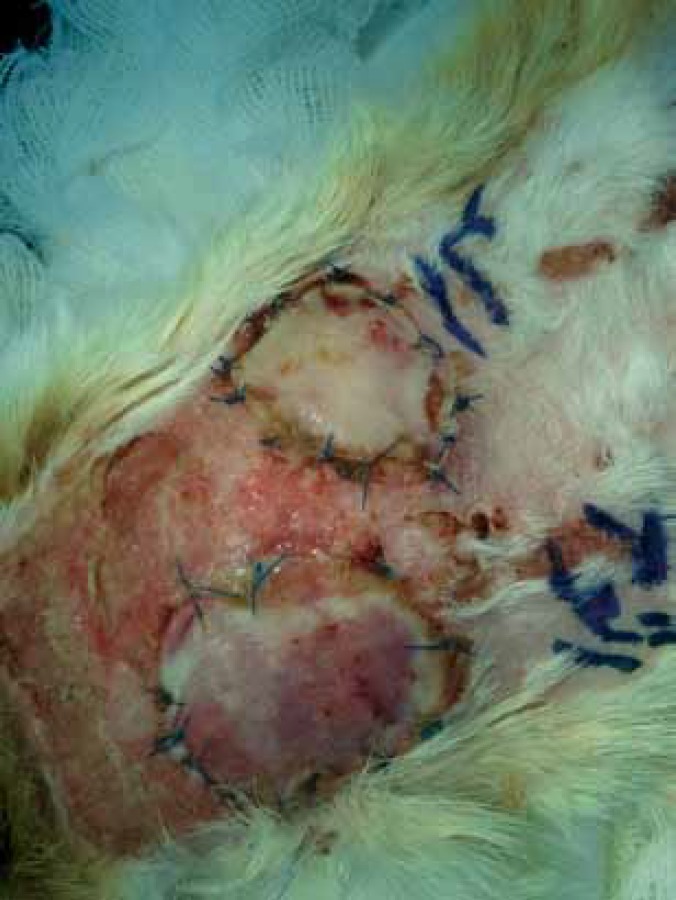
Skin allografts on the 3^rd^ day

**Figure 2:**
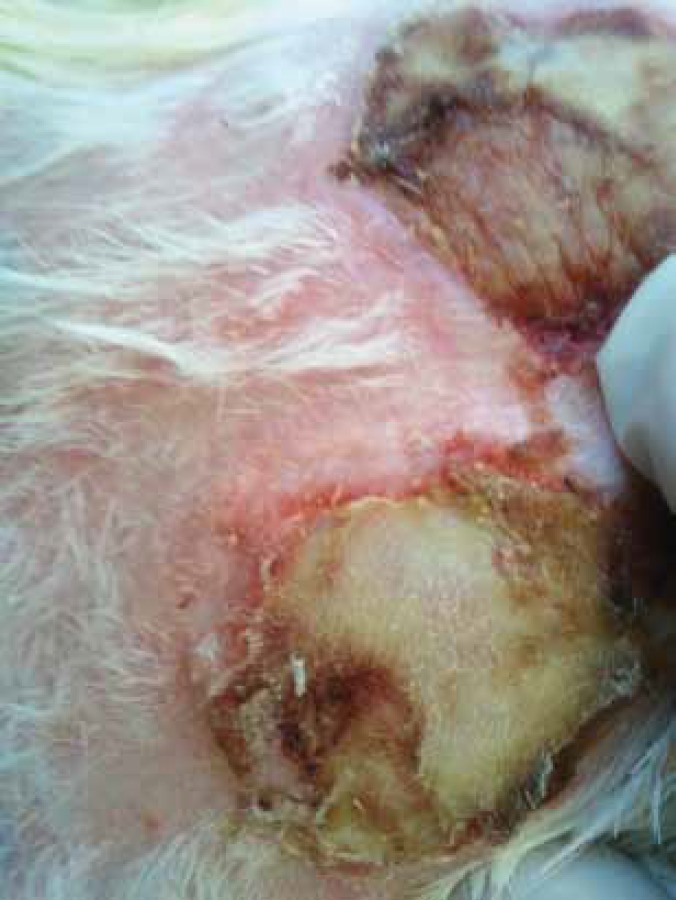
Skin allografts on the 13^th^ day

In the daily examination of rabbits, all grafts were separated from the base in 15–20 days. There was no difference between skin allografts from BDD *vs* cardiac-dead donors. In inspection, there were no difference between the two sides in terms of inflammation and hyperemia (Figs 1, 2).

There were no differences in the numbers of lymphocytes or Langerhans cells present in the skin obtained from the two sources of donors in histologic examination, one and two weeks of transplantation.

## DISCUSSION

The prerequisites of an ideal wound dressing are its resemblance to the normal skin, and its ability not to induce any inflammatory complications. Existence of a convenient means for its transportation is another important factor to be seriously considered. By freeze-drying or lyophilization, the skin can easily be preserved and transported for medical purposes. This study showed that the processing procedure for samples taken from BDDs does not differ from those obtained from cardiac dead donors and that the results were promising; it was not toxic to the animals.

Given that the need for skin allografts is continued, many experts are looking for increasing the source and overcoming logistical difficulties. Even looks extraordinary, Wang, *et al*, recommend accepting chronic HBV-infected skin donors due to its significant low probability of concurrent positive rates of HBV DNA in the serum and skin [11, 12]. The results of our study including other tissue banks, showed the promising results for future usage in patients. Having skin from BDDs enables us not only to increase the skin but also to decrease the number of discarded samples due to microbial contamination because of fully sophisticated laboratory checked organ donors and retrieval in sterile operating theater setting [13].
